# Expression of SIRT1–7 mRNA in Patients with Middle Ear Cholesteatoma

**DOI:** 10.3390/jcm15208195

**Published:** 2026-10-21

**Authors:** Jeon Gang Doo, Jae Min Lee, Minsuh Park, Hye Ok Kim, Minji Choi, Yoo Jin Cha, In Uk Kang, Sung Soo Kim, Seung Geun Yeo

**Affiliations:** 1 Department of Otorhinolaryngology—Head & Neck Surgery, Nowon Eulji Medical Center, Eulji University School of Medicine, Seoul 02447, Republic of Korea; riversoul@naver.com; 2 Department of Otorhinolaryngology—Head and Neck Surgery, College of Medicine, Kyung Hee University Medical Center, Kyung Hee University, Seoul 02447, Republic of Korea; jmlee3042@khu.ac.kr; 3 Department of Food & Nutrition, College of Human Ecology, Kyung Hee University, Seoul 02447, Republic of Korea; abcming@naver.com; 4 Clinical Research Institute, Kyung Hee University Medical Center, Seoul 02447, Republic of Korea; hyeokkim@khu.ac.kr (H.O.K.); a5033@khmc.or.kr (M.C.); 5 Department of Medicine, College of Medicine, Kyung Hee University, Seoul 02447, Republic of Korea; swj09161@khu.ac.kr; 6 Department of History, Kyung Hee University, Seoul 02447, Republic of Korea; kanginuk@khu.ac.kr; 7 Department of Biochemistry and Molecular Biology, College of Medicine, Kyung Hee University, Seoul 02447, Republic of Korea; sgskim@khu.ac.kr

**Keywords:** cholesteatoma, otitis media, sirtuins, *SIRT7*, oxidative stress, inflammation

## Abstract

**Background:** Middle ear cholesteatoma is a locally destructive lesion that can cause severe complications and cannot be effectively managed with medical treatment alone. Although sirtuins are crucial epigenetic regulators of cellular metabolism, oxidative stress, and inflammatory responses, their specific roles in middle ear cholesteatoma remain unexplored. This study aimed to investigate the expression profiles of sirtuins and their association with the pathogenesis and clinical characteristics of cholesteatoma. **Methods:** The relative mRNA expression levels of seven sirtuin isoforms (*SIRT1–7*) were evaluated using quantitative real-time PCR in cholesteatoma tissues and matched postauricular skin controls obtained from 60 patients undergoing cholesteatoma surgery. Expression differences were analyzed according to tissue type, patient sex, and frequency of surgery (initial versus revision). **Results:** Clinically, otorrhea was observed in 58.3% of the patients, whereas the bacterial culture positivity rate was 16.7%. Conductive hearing loss was the predominant audiologic configuration. *SIRT1–7* mRNA expression was detected in all cholesteatoma tissues, with *SIRT1* exhibiting the highest relative expression, followed by *SIRT4*, *SIRT3*, *SIRT6*, *SIRT7*, *SIRT5,* and *SIRT2*. Notably, only the expression of *SIRT7* was significantly downregulated in cholesteatoma tissues compared to postauricular skin controls (*p* = 0.002). No significant differences in *SIRT1–7* expression were observed according to patient sex or history of revision surgery (*p* > 0.05). **Conclusions:** The distinct expression profiles of *SIRT1–7* indicate their potential involvement in the pathogenesis of middle ear cholesteatoma. Although *SIRT7* mRNA expression was lower in cholesteatoma tissue than in postauricular skin, this exploratory finding requires validation using tissue-appropriate controls and functional studies.

## 1. Introduction

Middle ear cholesteatoma is a chronic condition characterized by the abnormal hyperproliferation of keratinized squamous epithelium and the subsequent progressive accumulation of keratin debris within the middle ear cavity [1,2]. Histologically, cholesteatoma is composed of three distinct layers: a matrix consisting of keratinizing stratified squamous epithelium, a perimatrix comprising subepithelial connective tissue, and the innermost cystic content containing accumulated keratin [3]. The perimatrix is heavily infiltrated by various inflammatory cells, including lymphocytes, histiocytes, plasma cells, and neutrophils, and its thickness directly correlates with the severity of the local inflammation and subsequent osteolytic activity [4]. Clinically, this continuous structural alteration provokes a persistent local infection and irreversible destruction of the adjacent bony structures, leading to conductive or sensorineural hearing loss and potentially fatal complications such as labyrinthine fistulas or intracranial infections [5,6].

While the precise mechanisms underlying the initiation and progression of cholesteatoma remain a subject of ongoing debate, it is widely accepted that chronic inflammation and oxidative stress play pivotal roles in its pathogenesis [7]. The continuous infiltration of immune cells in the perimatrix not only secretes a multitude of pro-inflammatory cytokines but also generates excessive reactive oxygen species (ROS). This disruption of redox homeostasis—oxidative stress—exacerbates tissue damage, promotes the abnormal hyperproliferation and differentiation of the epithelial matrix, and actively stimulates osteoclast-mediated bone resorption [1]. Therefore, understanding the molecular mechanisms that regulate this inflammatory and oxidative microenvironment is crucial for elucidating the etiology of middle ear cholesteatoma.

Sirtuins are a highly conserved family of nicotinamide adenine dinucleotide (NAD+)-dependent enzymes that function as fundamental metabolic sensors, directly linking cellular energy status to adaptive stress responses [8]. In mammals, seven sirtuin isoforms (SIRT1–7) have been identified, each possessing distinct subcellular localizations and specific substrate specificities, emerging as critical regulators of cellular homeostasis [9]. Crucially, sirtuins act as master regulators of redox homeostasis, intimately involved in mitigating oxidative stress, modulating inflammatory cascades, regulating mitochondrial function, and controlling cellular senescence and apoptosis [10]. By deacetylating various histone and non-histone proteins, including key transcription factors, sirtuins orchestrate cellular defense mechanisms against oxidative and genotoxic damage [11,12].

Given the significant involvement of sirtuins in antioxidant defense and immune regulation, it is highly plausible that altered expression of *SIRT1–7* is closely associated with the persistent inflammation and oxidative stress observed in cholesteatoma. However, a comprehensive analysis of the expression profiles of all seven sirtuin isoforms in middle ear cholesteatoma has not yet been conducted. Therefore, this study aimed to evaluate the mRNA expression levels of *SIRT1–7* in cholesteatoma tissues compared to postauricular skin controls, and to investigate their potential clinical implications in the pathogenesis of the disease.

## 2. Materials and Methods

### 2.1. Subjects

This retrospective observational study included 60 patients who visited the Otorhinolaryngology-Head and Neck Surgery clinic at a single tertiary care center. Middle ear cholesteatoma was defined by the presence of persistent symptoms (e.g., hearing loss, tympanic membrane perforation, otorrhea, tinnitus, and otalgia) lasting for more than three months, alongside lesions in the middle ear and mastoid cavity confirmed via temporal bone computed tomography (TBCT), and a definitive histopathological diagnosis of cholesteatoma obtained from preoperative or intraoperative biopsies. The presence of otorrhea was assessed using otoscopy or endoscopy, and pus cultures were obtained if discharge was present in the external auditory canal or middle ear. The presence and severity of hearing impairment were evaluated using pure-tone audiometry (PTA). Written informed consent was obtained from all patients or their legal guardians after a thorough explanation of the study’s purpose, granting prior permission for the use of clinical samples. Patients suspected of having acute otitis media, otitis media with effusion, non-cholesteatomatous chronic otitis media, tuberculous otitis media, head and neck anomalies, systemic diseases, or congenital/acquired immunodeficiency were excluded. Patient medical records were retrospectively reviewed to collect data on age, sex, symptom onset date, affected side, bacterial culture results, underlying diseases, PTA results, and history of revision surgery. This study was approved by the Institutional Review Board of Kyung Hee University Medical Center (IRB 2023-05-016, approved on 15 May 2023), and written informed consent was obtained from all subjects.

### 2.2. Bacterial Culture

Microbiological evaluations were conducted for patients exhibiting otorrhea. Following the cleaning of the external auditory canal to prevent contamination, otorrhea specimens were collected aseptically utilizing sterilized cotton swabs (Xomed Trace Products, Jacksonville, FL, USA) under otoscopic guidance. The swabs were immediately placed into Stuart transport medium. Specimens were then inoculated onto solid blood agar and liquid thioglycollate medium (Hangang, Gunpo, Republic of Korea) and incubated at 35 °C for 24 h. Bacterial colonies were identified using Gram staining and standard biochemical tests.

### 2.3. Evaluation of Level of Hearing Loss

Air-conduction (AC) and bone-conduction (BC) hearing thresholds were measured using PTA at the time of diagnosis. The hearing threshold was calculated using a weighted average of the 500, 1000, and 2000 Hz frequencies, with the 1000 Hz threshold assigned double weighting (a four-frequency pure-tone average method). Hearing loss was categorized as follows: sensorineural hearing loss (SNHL) was defined as a BC threshold ≥ 30 dB with an air-bone (AB) gap < 10 dB; conductive hearing loss (CHL) was defined as an AB gap ≥ 10 dB with a BC threshold < 25 dB. Cases of mixed hearing loss (BC threshold ≥ 25 dB and AB gap ≥ 10 dB) were grouped with the SNHL category for the purpose of this analysis [13].

### 2.4. RNA Extraction and Quantitative Real-Time PCR (RT-qPCR)

Total RNA was extracted from the patient tissue samples (cholesteatoma and matched postauricular skin controls, *n* = 60 pairs) using TRIzol reagent (Invitrogen, Carlsbad, CA, USA) according to the manufacturer’s protocol. The concentration and purity of the extracted RNA were assessed using a NanoDrop spectrophotometer (Thermo Fisher Scientific, Waltham, MA, USA), and only samples with an A260/A280 ratio greater than 1.8 were used for downstream applications. cDNA was synthesized from 1 μg of total RNA using oligo(dT)18 primers with the TOPscript™ RT DryMIX (RT200, Enzynomics, Daejeon, Republic of Korea). The primer sequences used in this study are listed in Table 1. Primer specificities were confirmed via melting curve analysis, and amplification efficiencies were validated using serial dilution standard curves (efficiencies ranging from 90% to 110%). Quantitative real-time PCR was performed on a StepOnePlus Real-Time PCR System (Applied Biosystems, Foster City, CA, USA) utilizing the SensiFAST SYBR Hi-ROX Kit (Bioline, London, UK). The PCR was conducted with 2 μL of cDNA in a 20 μL reaction mixture containing 10 μL of SensiFAST SYBR Hi-ROX Mix, 2 μL of primers (1 μL each of forward and reverse), and 6 μL of PCR-grade water. No-template controls were included in all PCR runs to monitor for contamination. The thermal cycling conditions consisted of an initial polymerase activation at 95 °C for 2 min, followed by 40 cycles of denaturation at 95 °C for 5 s and a combined annealing/extension step at 60 °C for 20 s. The relative mRNA expression of each target gene was normalized to that of β-actin using the 2^^-ΔCt^ method, where ΔCt = Ct(target gene) − Ct(*β-actin*) [14,15]. The endogenous reference gene, *β-actin*, was selected due to its highly stable Ct values and lack of significant differential expression across both tissue types in this cohort. The mRNA expression levels of *SIRT1–7* were compared between the cholesteatoma tissues and postauricular skin controls.

### 2.5. Statistical Analysis

Statistical analyses were performed using IBM SPSS Statistics for Windows, version 20.0 (IBM Corp., Armonk, NY, USA). Continuous variables are presented as the mean ± standard deviation (SD), and categorical variables as frequencies and percentages. The normality of continuous variables was assessed prior to comparative analyses. Because cholesteatoma and postauricular skin samples were obtained from the same patients, SIRT1–7 mRNA expression levels were compared between the matched tissue samples using a two-sided paired *t*-test. For comparisons between two independent groups, Student’s *t*-test was used for normally distributed variables, whereas the Mann–Whitney U test was used for non-normally distributed variables. Hearing status and the presence or absence of otorrhea were summarized descriptively to characterize the study population. All statistical tests were two-sided. To rigorously adjust for multiple comparisons across the seven sirtuin isoforms, a Bonferroni correction was applied, establishing a stringent threshold for statistical significance at *p* < 0.007 (calculated as 0.05/7).

## 3. Results

### 3.1. Demographics and Clinical Characteristics

A total of 60 patients with cholesteatoma were included in this study, with a mean age of 60.60 ± 13.07 years (range, 21–80 years). The cohort consisted of 27 males (45.0%) and 33 females (55.0%). Lesions were located in the right ear in 40 patients (66.7%) and the left ear in 20 patients (33.3%). Regarding clinical presentation, otorrhea was observed in 35 patients (58.3%). Bacterial cultures were positive in 10 patients (16.7%), with *Pseudomonas aeruginosa* being the most commonly detected strain, followed by methicillin-resistant *Staphylococcus aureus*, coagulase-negative *Staphylococcus*, *Staphylococcus aureus*, and *Achromobacter xylosoxidans*. Additionally, 25 patients (41.7%) underwent revision surgery, and 29 (48.3%) had a history of previous mastoidectomy. Audiometric evaluations revealed a mean pure-tone audiometry (PTA) threshold of 64.10 ± 24.50 dB for air conduction (AC) and 38.58 ± 20.03 dB for bone conduction (BC). Conductive hearing loss was the most prevalent type, observed in 38 patients (63.3%), followed by sensorineural hearing loss in 21 patients (35.0%), and normal hearing in 1 patient (1.7%) (Table 2).

### 3.2. Expression Profiles of SIRT1–7 mRNA in Cholesteatoma Tissues

**Figure 1:**
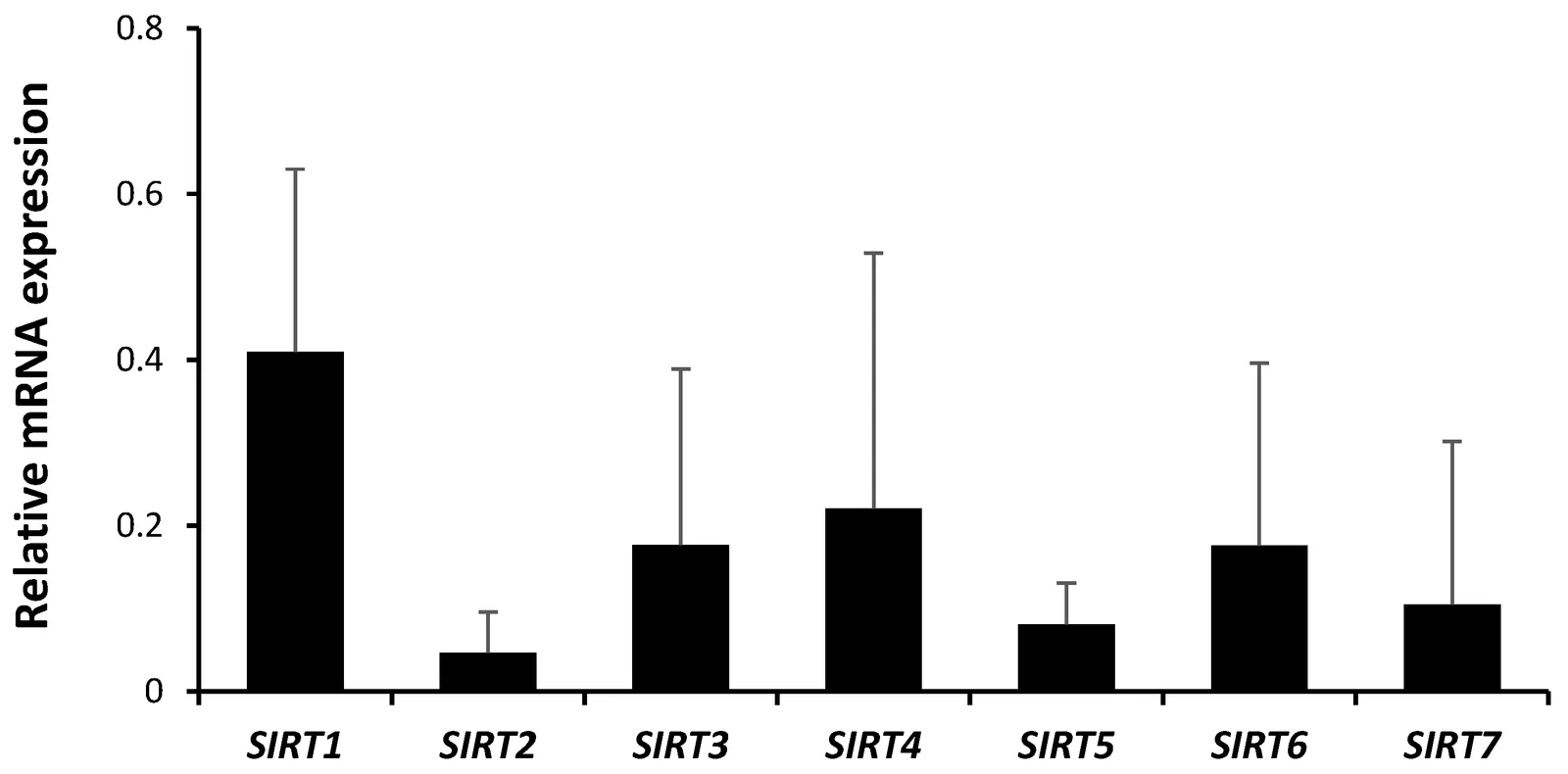
Relative mRNA expression levels of *SIRT1–7* in cholesteatoma tissues.

The expression of *SIRT1–7* mRNA was detected in the inflammatory middle ear tissues obtained from all 60 patients with cholesteatoma. Quantitative analysis revealed that *SIRT1* mRNA exhibited the highest relative expression level among the sirtuin family members. The relative expression levels, in descending order, were *SIRT1*, *SIRT4*, *SIRT3*, *SIRT6*, *SIRT7*, *SIRT5,* and *SIRT2* (Figure 1).

The mRNA expression profiles of the sirtuin family (*SIRT1–7*) were evaluated in inflammatory middle ear tissues obtained from patients with middle ear cholesteatoma (*n* = 60) using quantitative real-time PCR. The relative expression level of each target gene was calculated using the 2^^-ΔCt^ method and normalized to the endogenous reference gene, *β-actin*. Data are presented as the mean ± standard deviation (SD).

### 3.3. Comparison of SIRT1–7 mRNA Expression Between Postauricular Skin and Cholesteatoma Tissues

To investigate the differential expression of sirtuins, the mRNA levels of *SIRT1–7* in cholesteatoma tissues were compared with those in postauricular skin controls. The expression levels of *SIRT1*, *SIRT4*, and *SIRT6* showed an increasing trend in cholesteatoma tissues compared to the control skin tissues, whereas *SIRT2*, *SIRT3*, *SIRT5*, and *SIRT7* exhibited a decreasing trend. Notably, the relative mRNA expression of *SIRT7* was significantly downregulated in the cholesteatoma tissues compared to the postauricular skin controls (*p* = 0.002). Differences in the expression levels of the other sirtuin isoforms were not statistically significant (*p* > 0.05) (Table 3).

### 3.4. Association of SIRT1–7 mRNA Expression with Clinical Parameters

We evaluated whether the expression levels of SIRT1–7 mRNA in cholesteatoma tissues varied according to clinical parameters, specifically patient sex and surgical history. The analysis revealed no statistically significant differences in the expression profiles of any SIRT isoforms between male and female patients (*p* > 0.05) (Table 4). Furthermore, no significant differences were observed between the initial surgery and revision surgery groups (*p* > 0.05) (Table 5).

## 4. Discussion

Bone resorption in cholesteatoma was traditionally attributed to mechanical pressure from the cholesteatoma mass. However, subsequent studies demonstrated that osteolysis is driven by enzymatic and inflammatory mechanisms; inflammatory cells infiltrating the perimatrix, particularly histiocytes, secrete lytic enzymes, such as acid phosphatase and proteases, alongside pro-inflammatory cytokines, which collectively induce bone destruction [4,16]. Furthermore, cytokine-mediated inflammatory responses directly contribute to osteolysis. Macrophages, lymphocytes, epithelial cells, endothelial cells, and fibroblasts located near the eroded bone secrete diverse cytokines that induce vasodilation, increase vascular permeability, and promote granulation tissue proliferation, ultimately exacerbating bone destruction. Because middle ear cholesteatoma is characterized by chronic inflammation driven by recurrent bacterial infections, continuous infectious episodes trigger clinical symptoms such as chronic otorrhea and accelerate osteolytic complications. Specifically, previous studies have demonstrated that bacterial endotoxins, such as lipopolysaccharide, and the activation of innate immune receptors, including Toll-like receptors, strongly correlate with the severity of local inflammation and bone resorption in cholesteatoma [17,18]. The clinical behavior of cholesteatomatous disease is heterogeneous, and clinical presentation, anatomical extent, bone involvement, and management requirements may vary across disease entities. Motta et al. described the differential diagnosis and outpatient management of external auditory canal conditions, including external auditory canal cholesteatoma, and highlighted that bone erosion or extension may necessitate further imaging and surgical management [19]. Because external auditory canal cholesteatoma is anatomically distinct from middle-ear cholesteatoma, these findings provide clinical context regarding heterogeneity and management rather than direct evidence for the molecular mechanisms examined in the present study. The initiation and progression of cholesteatoma involve a complex interplay of inflammatory responses, oxidative stress, aberrant cell proliferation, and altered tissue homeostasis, which are closely associated with sirtuin functions. However, no previous study has comprehensively analyzed the expression profiles of *SIRT1–7* in cholesteatoma or evaluated their relationships with clinical characteristics. In the present study, we demonstrated that the relative mRNA expression of *SIRT7* was significantly downregulated in cholesteatoma tissues compared to postauricular skin controls.

Mammalian sirtuins are NAD^+^-dependent class III histone deacetylases (HDACs) and have seven distinct isoforms. Their substrate specificities are strictly dictated by their subcellular localizations: *SIRT1*, *SIRT6*, and *SIRT7* function primarily in the nucleus targeting histones, *SIRT2* regulates cytoskeletal dynamics in the cytoplasm, while *SIRT3*, *SIRT4*, and *SIRT5* modulate metabolic enzymes within mitochondria [20,21]. Considering these distinct spatial and functional partitions, the differential mRNA expression patterns of *SIRT1–7* observed in our study suggest that diverse cellular compartments in cholesteatoma respond differently to local oxidative and inflammatory stress. In the present study, the mRNA expression of all seven sirtuin isoforms (*SIRT1–7*) was detected in the cholesteatoma tissues of all 60 patients. *SIRT1* exhibited the highest relative expression level, followed in descending order by *SIRT4*, *SIRT3*, *SIRT6*, *SIRT7*, *SIRT5*, and *SIRT2*. When compared to the postauricular skin controls, the expression levels of *SIRT1*, *SIRT4*, and *SIRT6* demonstrated an increasing trend, whereas *SIRT2*, *SIRT3*, *SIRT5*, and *SIRT7* showed a decreasing trend. Notably, only the relative mRNA expression of *SIRT7* was significantly downregulated in the cholesteatoma tissues.

*SIRT1* is the most extensively studied isoform among the mammalian sirtuins. Homologous to the yeast Sir2 protein, which fundamentally regulates chromatin structure and transcriptional silencing [22], *SIRT1* functions as a histone deacetylase to induce epigenetic modifications. Furthermore, *SIRT1* targets diverse non-histone proteins, particularly major transcription factors such as p53 and NF-κB, to modulate critical cellular processes including cell survival, apoptosis, inflammatory responses, and DNA damage repair [23,24]. Other sirtuin isoforms also exert specific epigenetic and cell-cycle regulatory functions; for instance, *SIRT2* dictates chromatin condensation during mitosis via H4K16 deacetylation to maintain genomic stability [25]. In the present study, *SIRT1* exhibited the highest relative expression level among all sirtuin isoforms and demonstrated an increasing trend in cholesteatoma tissues compared to postauricular skin controls. These findings suggest that *SIRT1* may exert a regulatory role in the pathogenesis of chronic inflammation and bone resorption in cholesteatoma. However, the definitive contribution of *SIRT1* to the pathophysiology of cholesteatoma remains unclear, primarily because the observed increase lacked statistical significance and the comparison was conducted against postauricular skin rather than normal middle ear mucosa. Consequently, further investigations are required to elucidate its precise mechanisms in this disease.

However, the most critical finding of the present study is that, among the seven sirtuin isoforms, only the mRNA expression of *SIRT7* was significantly downregulated in cholesteatoma tissues compared to postauricular skin controls. To the best of our knowledge, no previous studies have investigated the specific role of *SIRT7* in the pathogenesis of cholesteatoma or otitis media. Although *SIRT7* remains relatively less characterized than other sirtuins, it primarily localizes to the nucleolus, where it regulates ribosomal DNA transcription, ribosome biogenesis, cell proliferation, genomic stability, and cellular stress responses [24]. In vivo studies utilizing *SIRT7* knockout mice have demonstrated that *SIRT7* deficiency increases cardiomyocyte apoptosis, reduces cellular resistance to oxidative and genotoxic stress, and induces severe inflammatory cardiomyopathy [26]. Cholesteatoma tissues constitute a complex microenvironment characterized by persistent inflammation, oxidative stress, and aberrant epithelial proliferation [27]. Given the complete absence of prior otological research on this isoform, the lower SIRT7 mRNA level observed in cholesteatoma tissue raises the possibility that SIRT7-related pathways may merit further investigation; however, the present data do not establish a functional or causal role for SIRT7 in cholesteatoma. Furthermore, based on the current data, it remains unclear whether the reduced SIRT7 expression is a primary causal factor driving the initiation and progression of cholesteatoma, or a secondary consequence of chronic inflammation and tissue damage. Consequently, further investigations are required to identify the specific cell types and spatial localization of *SIRT7* expression within cholesteatoma tissues.

Apart from *SIRT1* and *SIRT7*, the relative expression levels of *SIRT2*, *SIRT3*, and *SIRT5* demonstrated a decreasing trend, whereas *SIRT4* and *SIRT6* showed an increasing trend in cholesteatoma tissues; however, these changes lacked statistical significance. Given that these sirtuin isoforms broadly regulate cellular metabolism, aging, inflammatory and immune responses, oxidative stress, mitochondrial homeostasis, and apoptosis [21], they may also be involved in the pathogenesis of cholesteatoma. Nevertheless, the current study design is limited in determining their precise clinical relevance.

This study has several limitations. First, our analysis was restricted to the mRNA level. Because mRNA levels do not always correlate directly with protein expression or actual enzymatic activity, future studies utilizing immunohistochemistry, Western blot analysis, or proteomic profiling are required to validate these findings. Second, postauricular skin was utilized as the control rather than normal middle ear mucosa. Histologically, cholesteatoma consists of keratinizing stratified squamous epithelium, whereas the normal middle ear cavity is lined by ciliated pseudostratified columnar epithelium. Consequently, consistent with numerous previous molecular studies on cholesteatoma, matched postauricular skin (normal squamous epithelium) was selected to allow for a histologically matched comparison and to avoid confounding differences between distinct epithelial lineages. However, because the microenvironment of postauricular skin differs from that of the native middle ear, this approach precludes determining the baseline expression of SIRT1–7 mRNA in normal middle ear mucosa, and our findings should be interpreted with this limitation in mind. Third, the prophylactic administration of antibiotics for one week prior to surgery, intended to reduce inflammation, might have influenced the mRNA expression of *SIRT1–7*. Fourth, operative findings and bone erosion were not recorded using a standardized grading system in this retrospective study, and sufficiently complete patient-level data were not available for a reliable analysis. Fifth, although SIRT1–7 expression was detected in both skin and cholesteatoma tissues with distinct expression patterns, the direct biological correlation between these two disparate tissue types could not be fully established. Although hearing status and otorrhea were documented, their associations with SIRT1–7 mRNA expression were not statistically evaluated. This limits the clinical interpretation of the observed expression differences, particularly whether lower SIRT7 expression is associated with hearing impairment or the presence of otorrhea. The absence of these analyses should not be interpreted as evidence of no association. Future studies should specifically evaluate these relationships. Finally, we were unable to comprehensively analyze the differences in SIRT1–7 expression according to disease duration, clinical severity, extent of bone destruction, culture positivity status, presence of complications, or history of medical treatment. Furthermore, the present study was not statistically powered to establish definitive equivalence (i.e., a true lack of association) for the subgroup analyses based on patient sex or surgical history.

## 5. Conclusions

This exploratory study characterized SIRT1–7 mRNA expression in middle-ear cholesteatoma tissue relative to matched postauricular skin. Among the seven isoforms, SIRT7 mRNA expression was lower in cholesteatoma tissue in the current analysis, whereas the other isoforms did not show statistically significant differences. Because the control tissue was not normal middle-ear mucosa and protein expression and functional activity were not assessed, these findings do not establish a causal role for SIRT7 or the sirtuin family in cholesteatoma pathogenesis. Further studies using tissue-appropriate controls and functional assays are needed to determine the biological and clinical significance of this observation.

## Figures and Tables

**Table 1:** Primer sequences for real-time RT-PCR.

Gene	Sequences	Length (Base Pairs)
*SIRT1*	F: 5′-AGGCCACGGATAGGTCCATA-3′ R: 5′-GTGGAGGTATTGTTTCCGGC-3′	103
*SIRT2*	F: 5′-CTCTCACCCTCTGGAGACCC-3′ R: 5′-GCAGGAAGTCCATGTCTGCT-3′	107
*SIRT3*	F: 5′-GGCGGCAGGGACGATTATTA-3′ R: 5′-TGCCTCCACTTCCAACAACA-3′	83
*SIRT4*	F: 5′-CCGTAGAGCTGTGAGAGAATGA-3′ R: 5′-ACCCAATGGAGGCTTTCGAG-3′	109
*SIRT5*	F: 5′-CGGGGCCCAAGTAAATGGA-3′ R: 5′-GTCTCCACGGCTGCTTTCTA-3′	138
*SIRT6*	F: 5′-TCCCCGACTTCAGGGGTC-3′ R: 5′-AAGGTGGTGTCGAACTTGGG-3′	76
*SIRT7*	F: 5′-ACTTGGTCGTCTACACAGGC-3′ R: 5′-CTCAGGTCGGCAGCACTAAC-3′	121
*β-actin*	F: 5′-ACAGAGCCTCGCCTTTGC-3′ R: 5′-GATATCATCATCCATGGTGAGCTGG-3′	70

Abbreviations: SIRT, sirtuin; F, forward; R, reverse; *β-actin*, beta-actin.

**Table 2:** Demographic and clinical characteristics of patients with cholesteatoma.

Clinical Characteristic	Overall (*n* = 60)
Age (mean ± SD), years	60.60 ± 13.07 (range 21–80)
Sex, *n* (%)	
Male	27 (45.0%)
Female	33 (55.0%)
Affected side, *n* (%)	
Right	40 (66.7%)
Left	20 (33.3%)
Otorrhea-positive, *n* (%)	35 (58.3%)
Culture-positive, *n* (%)	10 (16.7%)
Culture-negative, *n* (%)	50 (83.3%)
Revision surgery, *n* (%)	25 (41.7%)
History of previous mastoidectomy, *n* (%)	29 (48.3%)
Audiologic configuration	
PTA (AC) of affected ear (mean ± SD), dB	64.10 ± 24.50
PTA (BC) of affected ear (mean ± SD), dB	38.58 ± 20.03
Hearing loss type	
Conductive hearing loss, *n* (%)	38 (63.3%)
Sensorineural hearing loss, *n* (%)	21 (35.0%)
Normal hearing, *n* (%)	1 (1.7%)

Values are presented as mean ± SD (range) or *n* (%). All audiometric values were obtained from the affected (operated) ear. Hearing loss type was determined in the affected ear; patients with mixed hearing loss were included in the sensorineural hearing loss group. PTA, pure tone audiometry; AC, air conduction; BC, bone conduction.

**Table 3:** Expression of mRNA encoding target genes in skin and cholesteatoma.

mRNA	Skin	Cholesteatoma	*p*-Value
*SIRT1*	0.349 ± 0.290	0.410 ± 0.220	0.397
*SIRT2*	0.060 ± 0.032	0.047 ± 0.049	0.174
*SIRT3*	0.261 ± 0.192	0.177 ± 0.212	0.182
*SIRT4*	0.170 ± 0.209	0.221 ± 0.308	0.870
*SIRT5*	0.143 ± 0.162	0.081 ± 0.050	0.874
*SIRT6*	0.060 ± 0.053	0.176 ± 0.220	0.062
***SIRT7***	**0.247 ± 0.214**	**0.105 ± 0.197**	**0.002**

Values are presented as the mean ± standard deviation (SD). *p*-values were calculated using two-sided paired *t*-test comparing matched cholesteatoma and postauricular skin samples from the same patient (*n* = 60 pairs). Bold values indicate statistical significance at the Bonferroni-corrected threshold of *p* < 0.05/7 (approximately 0.0071).

**Table 4:** Association between expression of mRNA encoding target genes in cholesteatoma samples and patient sex.

mRNA	Cholesteatoma		
	Male	Female	*p*-Value
*SIRT1*	0.433 ± 0.237	0.373 ± 0.231	1.000
*SIRT2*	0.048 ± 0.066	0.047 ± 0.036	0.686
*SIRT3*	0.219 ± 0.273	0.127 ± 0.115	0.836
*SIRT4*	0.339 ± 0.369	0.063 ± 0.066	0.301
*SIRT5*	0.083 ± 0.043	0.080 ± 0.061	0.779
*SIRT6*	0.166 ± 0.176	0.189 ± 0.281	0.758
*SIRT7*	0.122 ± 0.251	0.083 ± 0.110	0.758

Values are presented as the mean ± standard deviation (SD). *p*-values were calculated using the two-sided Mann–Whitney U test.

**Table 5:** Association between expression of mRNA encoding target genes in cholesteatoma samples and frequency of surgery.

mRNA	Cholesteatoma		
	Initial	Revision	*p*-Value
*SIRT1*	0.442 ± 0.251	0.358 ± 0.190	0.571
*SIRT2*	0.075 ± 0.059	0.019 ± 0.009	0.343
*SIRT3*	0.247 ± 0.290	0.116 ± 0.102	0.534
*SIRT4*	0.274 ± 0.334	0.181 ± 0.305	0.518
*SIRT5*	0.077 ± 0.042	0.087 ± 0.062	0.779
*SIRT6*	0.226 ± 0.255	0.126 ± 0.182	0.195
*SIRT7*	0.087 ± 0.103	0.123 ± 0.268	0.574

Values are presented as the mean ± standard deviation (SD). *p*-values were calculated using the two-sided Mann–Whitney U test.

## Data Availability

The original contributions presented in this study are included in the article. Further inquiries can be directed to the corresponding author.
